# MitCHAP-60 and Hereditary Spastic Paraplegia SPG-13 Arise from an Inactive hsp60 Chaperonin that Fails to Fold the ATP Synthase β-Subunit

**DOI:** 10.1038/s41598-019-48762-5

**Published:** 2019-08-23

**Authors:** Jinliang Wang, Adrian S. Enriquez, Jihui Li, Alejandro Rodriguez, Bianka Holguin, Daniel Von Salzen, Jay M. Bhatt, Ricardo A. Bernal

**Affiliations:** University of Texas at El Paso, Department of Chemistry and Biochemistry, 500 West University Ave., El Paso, Texas 79968 USA

**Keywords:** Neurochemistry, Chaperones

## Abstract

The human mitochondrial heat shock protein 60 (hsp60) is a tetradecameric chaperonin that folds proteins in the mitochondrial matrix. An hsp60 D3G mutation leads to MitCHAP-60, an early onset neurodegenerative disease while hsp60 V72I has been linked to SPG13, a form of hereditary spastic paraplegia. Previous studies have suggested that these mutations impair the protein folding activity of hsp60 complexes but the detailed mechanism by which these mutations lead the neuromuscular diseases remains unknown. It is known, is that the β-subunit of the human mitochondrial ATP synthase co-immunoprecipitates with hsp60 indicating that the β-subunit is likely a substrate for the chaperonin. Therefore, we hypothesized that hsp60 mutations cause misfolding of proteins that are critical for aerobic respiration. Negative-stain electron microscopy and DLS results suggest that the D3G and V72I complexes fall apart when treated with ATP or ADP and are therefore unable to fold denatured substrates such as α-lactalbumin, malate dehydrogenase (MDH), and the β-subunit of ATP synthase in *in-vitro* protein-folding assays. These data suggests that hsp60 plays a crucial role in folding important players in aerobic respiration such as the β-subunit of the ATP synthase. The hsp60 mutations D3G and V72I impair its ability to fold mitochondrial substrates leading to abnormal ATP synthesis and the development of the MitCHAP-60 and SPG13 neuromuscular degenerative disorders.

## Introduction

Misfolded proteins are associated with many well-known human diseases such as Alzheimer’s disease, Parkinson’s disease, cancer, cystic fibrosis, and other neuropathies, highlighting the importance of protein folding in human health^1–3^. Protein folding is an important cellular process that depends on proteins reaching their native and fully functional three-dimensional structure^4^. Cells have evolved macromolecular machines called chaperonins that specialize in maintaining cellular homeostasis by assisting proteins that have become misfolded^5–7^. Protection provided by chaperonins is critical to support life since deletion of chaperonin genes from bacteria and yeast prove lethal^8–10^. Our studies focus on two neurodegenerative disorders termed SPG13 and MitCHAP-60 that have been linked to mutations in the human chaperonin, heat shock protein 60 (hsp60). Although both disorders are neurodegenerative, they differ in the clinical symptoms presented and mode of inheritance.

SPG13 is a member of hereditary spastic paraplegia (HSP) group of neurodegenerative diseases that are characterized by progressive weakness and spasticity (rigidity) in the lower extremities^11,12^. Patients with advanced HSP diseases show additional symptoms including impaired vision, deafness, and cognitive impairment. HSP diseases are neuropathologically characterized by degradation of the longest axons in the motor and sensory systems in the spinal cord^13,14^. To date, 72 spastic gait disease-loci and 55 spastic paraplegia genes (SPG) have been identified in humans^12^. Our study focuses on the HSP disease SPG13, which is a dominantly inherited form that presents at any age and is characterized by gait disturbances owing to progressive spasticity and weakness of the lower limbs^15,16^.

MitCHAP-60 is an early-onset neurodegenerative disease inherited in an autosomal-recessive pattern characterized by neuronal hypomyelination and leukodystrophy (brain white matter degradation)^16^. Patients show symptoms of nystagmus (involuntary eye movement) and psychomotor developmental delays during the first months of life that later progress to muscle weakness and limb spasticity (rigidity). MitCHAP-60 usually leads to death within the first two decades of life^16^. Both SPG13 and MitCHAP-60 have been linked to point mutations in hsp60. A D3G hsp60 mutation is a missense mutation that has been linked to MitCHAP-60 while a V72I mutation results in SPG13^16–19^. Previous studies have demonstrated that the disease-causing D3G and V72I mutations reduce the chaperonin ATP hydrolysis activity and subsequent protein folding ability of the resultant hsp60 complexes both *in-vitro* and *in-vivo*^17,19^. In this study, we investigated the molecular mechanism by which the hsp60 mutations lead to the MitCHAP-60 and SPG13.

The protein-folding machinery in the human mitochondria consists of a human mitochondrial heat shock protein (hsp60) and its co-chaperonin, human mitochondrial heat shock protein 10 (hsp10). They (hsp60/10 hereon) predominantly fold proteins in the mitochondrial matrix but have also been found in extra-mitochondrial locations^10,20–22^. Chaperonins assist in the folding of proteins in an ATP-dependent manner. They undergo ATP-driven conformational rearrangements to provide conditions that guide the folding of nascent or misfolded proteins and prevent aggregation leading to cell death.

It is difficult to isolate tetradecameric double-ring hsp60/10 complexes due to the labile nature of hsp60/10 complexes *in-vitro*. Most of the functional details known about the hsp60/10 system have been inferred from the catalytic cycle of groEL/groES, the bacterial homolog of hsp60/10 and from the bacteriophage chaperonin φEL^23–26^. Previous studies have demonstrated that the disease-causing D3G and V72I mutations reduce the chaperonin ATP hydrolysis activity and subsequent protein folding ability of the resultant hsp60 complexes both *in-vitro* and *in-vivo*^17,19^. However, due to the labile nature of hsp60/10 complexes, these studies had to reconstitute the expressed hsp60 proteins to form tetradecameric rings. Recently, we were able to purify fully assembled and functional human hsp60/10 complexes and reported that in the absence of nucleotide and substrate, hsp60 forms a stable tetradecameric conformation^27^. In this study, we isolated hsp60/10 complexes to elucidate the biochemical and structural basis for the aforementioned neurodegenerative disorders. Here, we show that hsp60 plays a crucial role in folding important players in aerobic respiration such as the β-subunit of the ATP synthase. Negative-stain electron microscopy along with DLS results suggest that the D3G and V72I complexes fall apart when treated with ATP or ADP and impair their ability to fold mitochondrial substrate proteins.

## Methods

### Cloning

The genes encoding the full-length wild type hsp60 (HspD1) and Hsp10 (HspE1) with the mitochondrial targeting sequence were cloned into the pET-30a expression vector system (EMD Millipore). Mature wild type hsp60 and the D3G and V72I mutations were cloned into pET-22b. We designate the mutations as D3G and V72I since that is the position in which they appear in the mature hsp60 protein that lacks the mitochondrial signaling peptide. All the primers were synthesized by IDT. All the constructs were sequence verified by the Genomic Analysis Core Facility at the University of Texas at El Paso. pET-26b vectors encoding the human ATP synthase F_1_ α-subunit (ATP5F1A) and human ATP synthase F_1_ β-subunit (ATP5F1B) were purchased from GenScript.

### Protein expression and purification

Unless otherwise stated, all chemicals, antibiotics, and growth media were purchased from Sigma-Aldrich. *E*. *coli* BL21 (DE3) cells were purchased from New England Biolabs. Hsp60 and hsp10 were expressed and purified as described in Enriquez *et al*.^27^. Briefly, the proteins were expressed in *E*. *coli* BL21 (DE3) cells cultured in 2xTY medium at 37 °C. Cells were induced with IPTG at 30 °C for 4 hours and were subsequently harvested by centrifugation at 5000 × g for 30 minutes and lysed in a buffer containing 50 mM HEPES pH 7.5, 50 mM EDTA, 0.02% NaN_3_. The cells were then lysed by treatment with hen egg white lysozyme and multiple freeze/thaw cycles. The viscous lysate was treated with porcine liver DNase and 100 mM MgCl_2_ to degrade DNA. Crude lysates were then treated with saturated ammonium sulfate to a final concentration of 50% (v/v) to precipitate the recombinant protein. Recombinant hsp60 and hsp10 were subsequently purified independently using anion-exchange and size-exclusion column chromatography (NGC Chromatography Systems, Bio-Rad) using chromatography buffer containing 50 mM HEPES, pH 7.5, 5 mM EDTA, 150 mM NaCl, and 0.02% NaN_3_.

The ATPase synthase α- and β- subunits were purified as described in Miwa and Yoshida, 1989^28^. The proteins were expressed in *E*. *coli* BL21 (DE3) cells cultured in 2xTY medium at 37 °C and induced with IPTG for four hours at 30 °C. Cells were spun as described above, lysed, and crude lysates were purified with saturated ammonium sulfate added to a final concentration of 75% (v/v). Proteins were purified by ion-exchange and size-exclusion chromatography using chromatography buffer containing 50 mM HEPES buffer, pH 7.0, 5 mM EDTA, 200 mM Na_2_SO_4_. SDS-PAGE and bicinchoninic acid (BCA) protein assay were utilized to estimate sample homogeneity and the concentrations of all purified proteins, respectively.

### Chaperonin ATPase activity assays

The ATPase activity assay was performed using the EnzChek Phosphatase Assay Kit (Molecular Probes, Leiden, The Netherlands) which measures the inorganic phosphate released from ATP hydrolysis during protein folding enzymatic reactions. The substrate α-lactalbumin was denatured with the addition of 0.5 mM EDTA and 50 mM DTT. The resultant mixture was heated to 98 °C for 5 minutes and allowed to cool before a 10-minute incubation period with 200 μM MESG, 0.2 units Purine Nucleoside Phosphorylase (PNPase) and the kit reaction buffer. Next, 200 μM ATP and 200 μM MgCl_2_ were added to a 100 μL reaction followed by addition of 1 μM hsp60 and 2 μM hsp10. A continuous colorimetric measurement at 360 nm was initiated immediately after the addition of hsp60 and hsp10.

Assays to refold the β-subunit of the ATP synthase were performed as follows. The purified β-subunit was thermally denatured by incubating at 55 °C for 20 min^29^. A reaction was set up with 200 μM MESG, 0.2 U PNPase, 200 μM ATP, 200 μM MgCl_2_, 1 μM hsp60, 2 μM hsp10, 1 μM α- subunit, and 1 μM denatured β- subunit. Next, the reaction buffer was added to yield a final volume of 100 μL. A continuous colorimetric read at 360 nm was initiated to monitor the rate of ATP hydrolysis.

### MDH refolding assay

Malate dehydrogenase (MDH) at a concentration of 62.5 nM was denatured in 10 mM HCl for 1 hour at room temperature and diluted 1:50 into a buffer containing 0.1 M Tris-HCl pH 7.4, 7 mM KCl, 7 mM MgCl_2_, 10 mM DTT, along with 1.3 μM hsp60, 2.6 μM hsp10, and 0.5 mM ATP. After 5 mins of incubation at 37 °C, the Malate Dehydrogenase Assay Kit (Sigma, MAK196) was used to measure the renatured malate dehydrogenase activity. The MDH enzymatic activity was determined by measuring the absorbance at 450 nm.

### Negative stain electron microscopy and single particle reconstruction

Purified chaperonin samples at a concentration of 0.2 mg/ml were applied to continuous carbon film with 400-mesh copper grids that were previously glow discharged for 30 seconds. Excess protein solution was wicked off with Whatman-1 filter paper and grids were stained with 2% methylamine tungstate and 2% uranyl acetate. The negative stain imaging was performed using a JEOL 3200FS transmission electron microscope operated at 300 kV and the images were collected between 0.3–2.5 μm under-focus and at a magnification of 138,000X using a Gatan UltraScan CCD camera. The pixel size calculated for the data set was 1.09 Å/pixel.

We collected data under the following conditions to capture the major nucleotide-dependent conformations within the hsp60/10 protein folding cycle. For the ATP-bound conformation (without hsp10), 2 mM ATP was added to hsp60. For the ATP-bound conformations in the presence of hsp10, 100 mM ATP was added to hsp60 and hsp10 (2:1 molar ratio). To obtain the ADP conformation, 2 mM Mg-ADP was mixed with hsp60 and hsp10 (1:1 molar ratio). The purified proteins with incubated with the nucleotides at room temperature for approximately five minutes before they were applied to the grids. A total of 583 particles were picked from the negative stained micrographs using EMAN2^30^. CTF correction, reference-free class averaging, initial model building, and single particle reconstruction were all performed in EMAN2^31^. Visualization and figures were generated using UCSF’s Chimera^32^.

Due to the heterogeneity in the hsp60/10 ATP-conformation dataset, a slightly different approach was utilized to separate the particles corresponding to the bullet and football complexes. The reference-free class averages that corresponded to the side-views of the two complexes were separated and individually used to generate two low resolution initial models. The two models were then used as references for the EMAN2 version of multi-refine to separate the particles into two sub-sets. The total data-set contained 3203 particles that were split into sub-sets containing 1654 (bullet) and 1536 (football) particles. Once the particles were split, single refinements were performed for both data-sets. C7 symmetry was used for the bullet refinement due to the different conformational states of the two rings^33^. The bullet reconstruction converged to a resolution of approximately 17 Å. D7 symmetry was used for the refinement of the football complex that converged to approximately 20 Å. The football complex generated in EMAN2 served as an initial model for additional refinements in RELION2 to reduce the amount of noise in the 3-D reconstruction^30,34^. For the hsp60 D3G and V72I APO conformations, 3144 and 2491 particles were picked form the negative stained micrographs, respectively. CTF correction, reference-free class averaging, initial model building, and single particle reconstruction were all performed in EMAN2. D7 symmetry was applied for the refinement using Eman2.

### Dynamic light scattering (DLS)

All experiments were performed on a Malvern Zetasizer Nano ZS instrument. For the ATP-bound conformation, 1 µM hsp60 (wild type or mutant) was added to 2 µM hsp10, 100 mM ATP, and 100 mM MgCl_2_. For the ADP-bound conformation, 1 µM hsp60 (wild type or mutant) was added to 2 µM hsp10, 2 mM ADP. The samples were incubated for 5 minutes at 37 °C upon addition of nucleotides. The excess unbound nucleotides were removed with desalting spin columns (Thermo Scientific). All the data are reported as percent volume to normalize the measurement with the amount of protein at each size. 180 measurements were averaged together to give a statistically significant size distribution that describes the hydrodynamic diameter of the complexes.

### Ethics approval

Ethics approval was not necessary for the study since standard procedures and safety measures were employed while using *E*. *coli* BL21 (DE3) bacterial cells (BSL-level 1 organism) that adhere with the institutional guidelines for Biosafety in research laboratories.

## Results

### Mutants have diminished protein-folding activity

**Figure 1:**
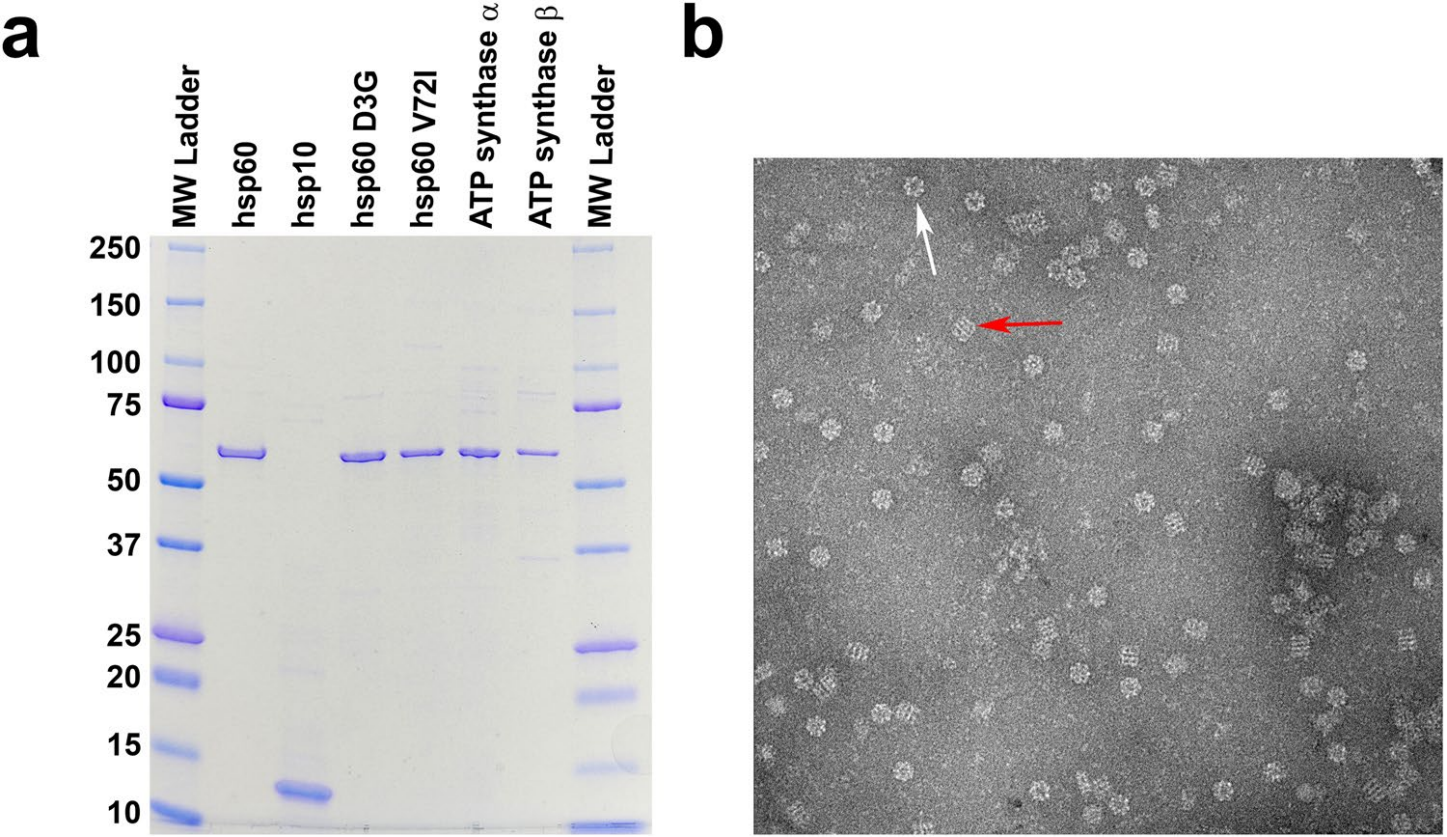
Purified chaperonin, mutants, and substrates. (**a**) SDS-PAGE of all the expressed and purified recombinant proteins used in our studies. The α and β subunits of the Human F_1_ ATP synthase appear to be the same size as the hsp60. Hsp10 runs anomalously as a 12 kDa protein. (**b**) A negative stain electron micrograph of hsp60 illustrating the formation of tetradecamers. The red arrow highlights a side-view of a tetradecamer and the white arrow points to an end view.

**Figure 2:**
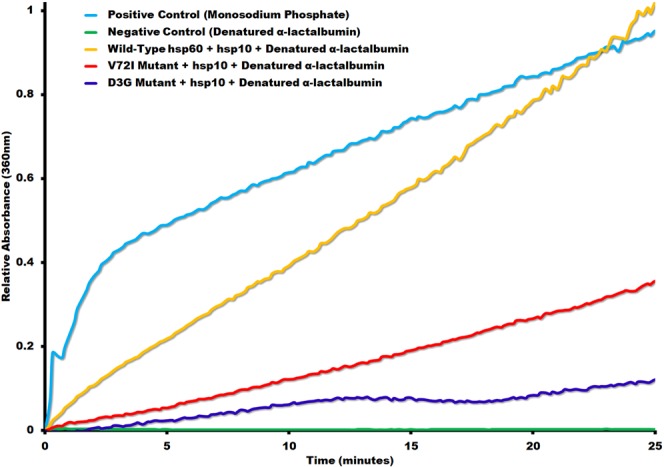
α-lactalbumin protein folding assay. ATPase activity of Recombinant Human Mitochondrial **wild-type** hsp60/hsp10, *V72I* mutant/hsp10 and D3G mutant/hsp10 in the presence of denatured α-lactalbumin substrate was measured by EnzChek Phosphate Assay. The assay measures released inorganic phosphate (P_i_) in the absorbance at 360 nm as a result of ATP hydrolysis by the chaperonin. The phosphate assay is generated using the monosodium phosphate as the ***positive control***. The **negative control** measures the ATPase activity of the denatured α-lactalbumin in the absence of chaperonin.

Human heat shock proteins (wild type hsp60/10 & mutants) and human F_1_ ATP synthase subunits (α and β) were expressed in *E*. *coli* and each purified to near homogeneity as described in the methods section. After each round of purification, the purity of all the proteins was estimated by SDS page analysis and determined to be near homogeneity (Fig. 1). Negative stain electron microscopy was done on freshly purified wild type hsp60/10 protein without nucleotide to make sure complexes were formed correctly (Fig. 1b). Chaperonin protein-folding activity was confirmed before proceeding with any experiments to make certain that we had biologically relevant complexes. Wild type hsp60/10 can effectively fold α-lactalbumin while the mutants had significantly diminished activity (Fig. 2). These results agree with previous reports that have studied the *in-vitro* catalytic activity of mutant hsp60 complexes^17,19^. Our results confirm their finding, albeit in a more biologically relevant system, since our hsp60 proteins were purified as fully assembled complexes and not as single-rings or monomers that had to be reconstituted to form functional tetradecamers.

### Architecture of the wild type hsp60/10 complex in the absence of substrate

**Figure 3:**
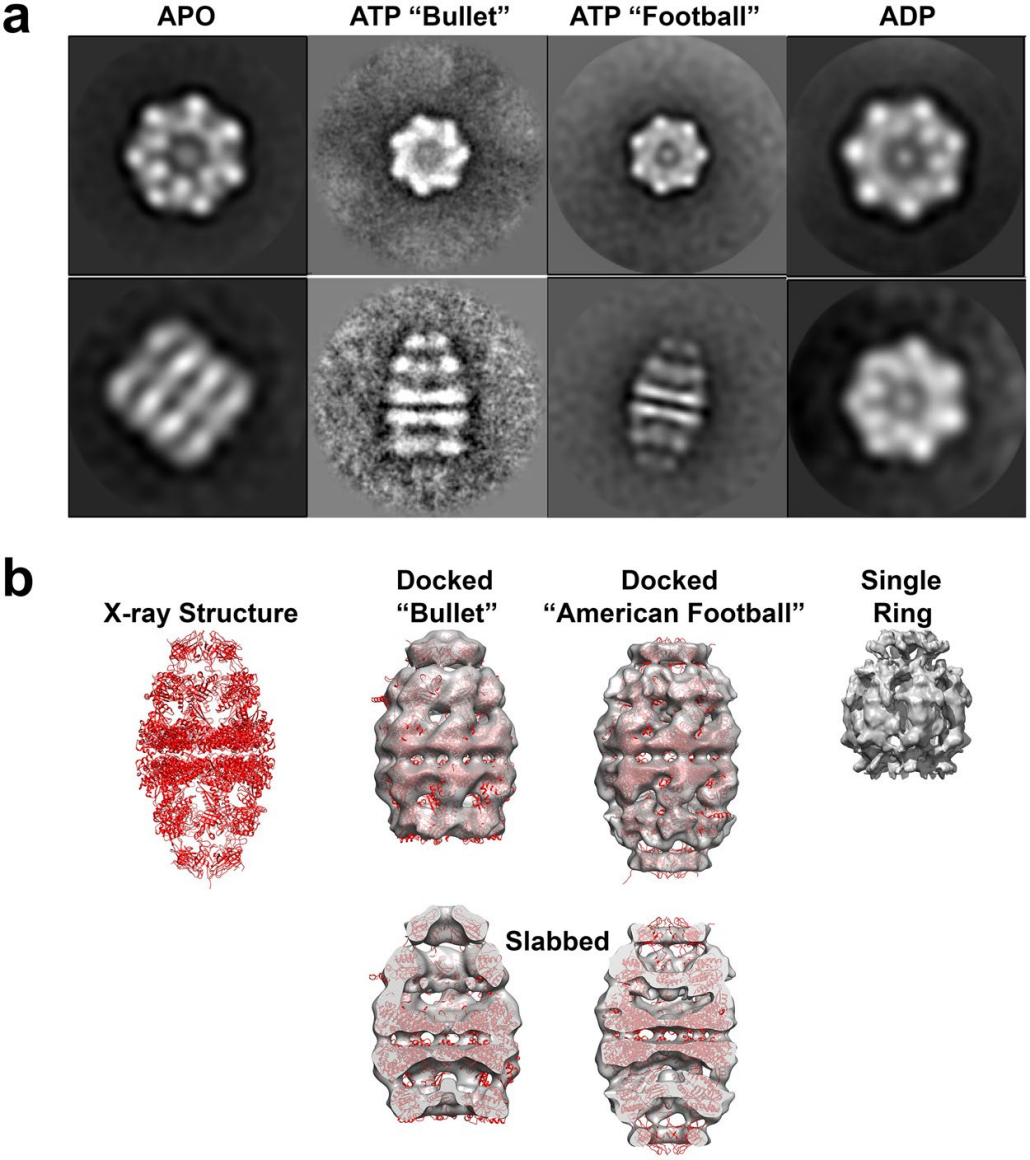
Negative stain reconstruction results. (**a**) Class averages of top views and side views below the top view for each of the four different conformations. The APO conformation has no nucleotide added to the purified protein. The “Bullet” and “Football” conformations are formed after the addition of different amounts of ATP, 2 mM and 100 mM respectively. (**b**) The published X-ray structure of the “Football” conformation is added as a reference for the negative stain reconstructions of the bullet and football (gray density) with the X-ray coordinates fitted into said reconstructions. There is no X-ray structure deposited for the single-ring ADP conformation.

**Figure 4:**
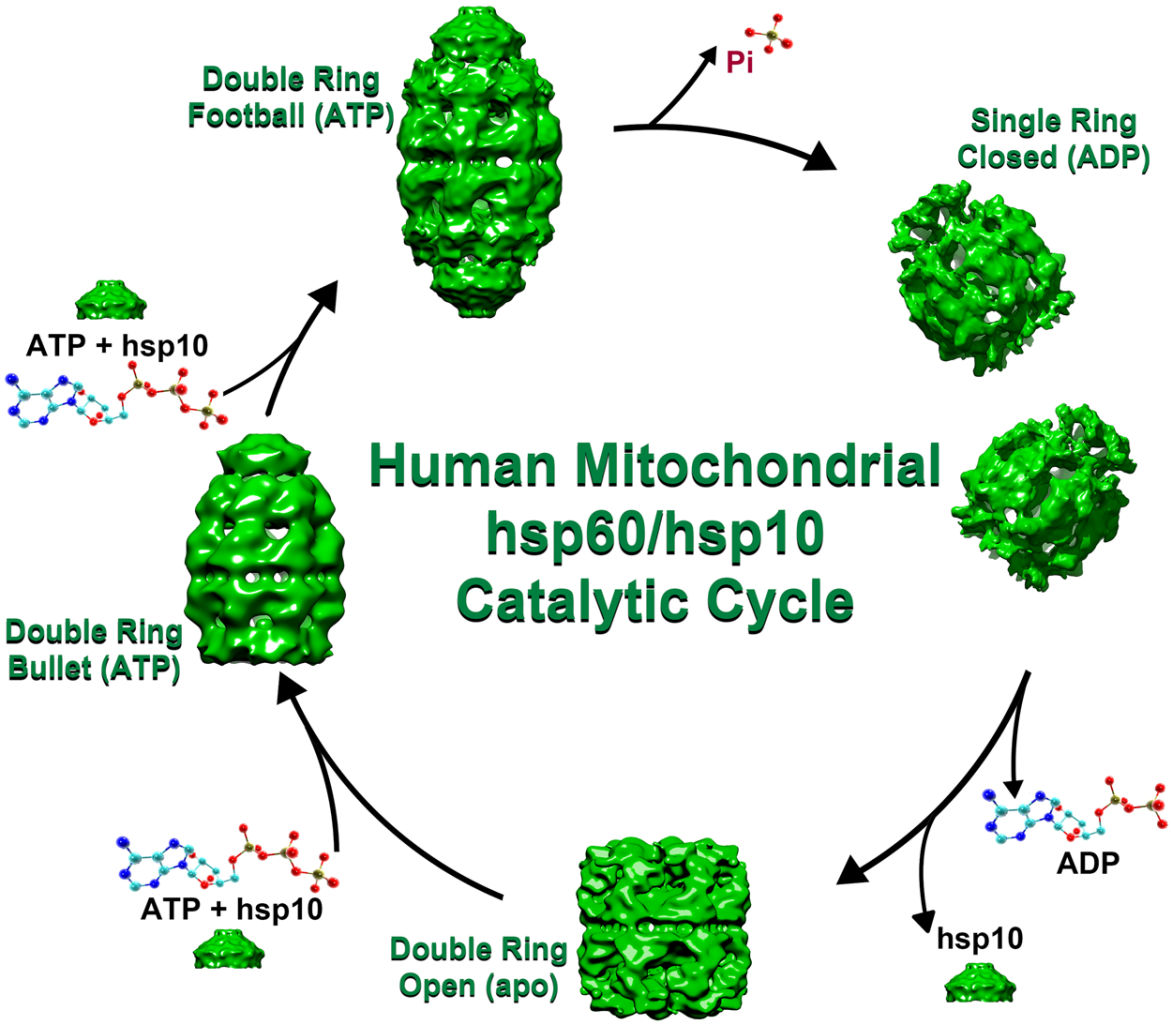
Proposed protein-folding intermediates along the catalytic pathway. The process begins with a self-assembled tetradecamer (Double-Ring) in the APO conformation. Binding of ATP induces the recruitment of both substrate (not shown) and the co-chaperonin hsp10 to produce the bullet conformation equivalent to the conformation seen when groEL hydrolyzes ATP to ADP. The addition of more ATP produces the football conformation with ATP bound to all 14 subunits and hsp10 bound to both ends. Upon ATP hydrolysis, the two rings separate to form an expanded single-ring conformation. Removal of ADP releases hsp10 and the substrate (not shown). The two rings then come back together to form the double-ring APO conformation once again.

The wild type mitochondrial hsp60/10 chaperonin utilizes the energy derived from ATP hydrolysis to undergo conformational changes that drive the protein-folding reaction. In the absence of substrate, the chaperonin complex stalls and does not progress along the protein-folding pathway until substrate is added^26^. We exploited this behavior to obtain near homogeneous conformations after the addition of nucleotide. Negative-stain electron microscopy on purified wild type protein without the addition of nucleotide resulted in double-rings that did not bind to hsp10 (Figs 1b and 3a). This was termed the APO conformation. Upon addition of 2 mM ATP to the same sample, the conformation changes to a double-ring structure with hsp10 bound to one of the rings (“bullet” structure, Fig. 3a). Addition of a higher concentration of ATP (100 mM) results in a conformation that is shifted from hsp10 binding to only one ring to binding on both rings (“American Football”, Fig. 3a). These bullet and American football conformations have been previously observed by other labs as well^35^. The addition of ADP mimics the ATP hydrolyzed state that produces a conformational state that induces ring separation resulting in single-rings of hsp60 with hsp10 bound (Fig. 3a). Negative-stain single-particle image processing resulted in low resolution reconstructions that better illustrate the conformational states as dictated by the nucleotide binding pocket. The X-ray structure of a chimeric complex of the mutant human mitochondrial Hsp60^E321K^ with mouse mitochondrial Hsp10 (PDB 4PJ1) was fit into the reconstructions for structural validation (Fig. 3b)^36^. No fitting of the single ring was performed because there is no X-ray structure of the single-ring. Based on the reconstructions of different conformations we observed under the electron microscope, we postulate a likely protein-folding cycle with the various intermediates along the catalytic pathway (Fig. 4). This is further elaborated in the discussion section

### Architecture of the mutant hsp60/10 complexes

**Figure 5:**
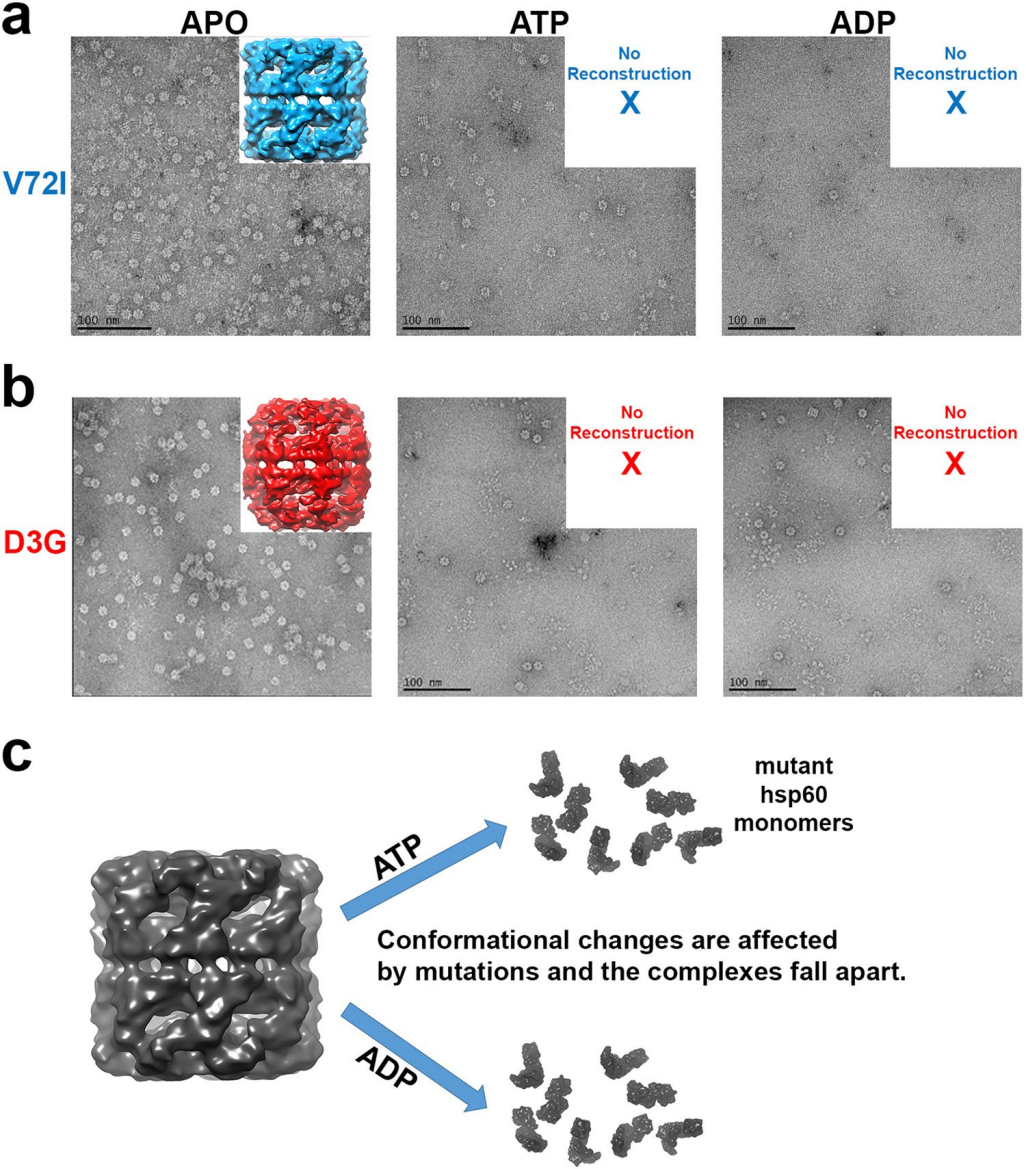
Negative stain reconstruction of mutant hsp60. (**a**) The V72I mutant resulted in an APO reconstruction that has a square appearance when viewed from the side. Upon the addition of nucleotide (either ATP or ADP), the majority of the chaperonin complexes fell apart. Consequently, no reconstruction was possible. (**b**) The D3G mutant also resulted in an APO reconstruction but it had a more rounded appearance when compared with the V72I mutant reconstruction. Just as with the V72I mutation, the addition of nucleotide caused the majority of the mutant chaperonin to fall apart. (**c**) The inability to undergo conformational changes upon nucleotide addition results in both mutant chaperonins to fall apart.

A similar approach as the wild type chaperonin was taken for the D3G and V72I mutant complexes. Negative stain raw micrographs of the two mutants show that just like the wild type chaperonin, each of the mutants can form double-ring complexes that do not bind hsp10 in the absence of substrate and nucleotide (Fig. 5a, APO conformation). Addition of either ATP or ADP resulted in mutant chaperonin complexes that fell apart (Fig. 5c). Data that support this observation include negative stain electron micrographs that illustrate very few intact chaperonins in a background of smaller monomeric proteins. To back this up, samples were measured using dynamic light scattering where it was observed that sample without nucleotide was of a size considerably larger than after the addition of either ADP or ATP. Note that this shift in size could be observed by the addition of the nucleotide directly to the cuvette containing the sample being measured. Negative-stain electron microscope data for low resolution three-dimensional reconstructions was only obtained from the APO conformation. A reconstruction of the mutants in the presence of nucleotide was not possible due to the heterogeneity and low numbers of individual mutant chaperonin complexes. Parnas *et al*. 2009 had previously reported that the D3G mutation leads to destabilization of the reconstituted hsp60 complexes using crosslinking experiments. Our study is the first to directly verify the oligomeric status of the mutant D3G and V72I hsp60 complexes using electron microscopy and DLS (see below) in the absence and presence of nucleotides ATP and ADP.

### Biochemical activity assays

**Figure 6:**
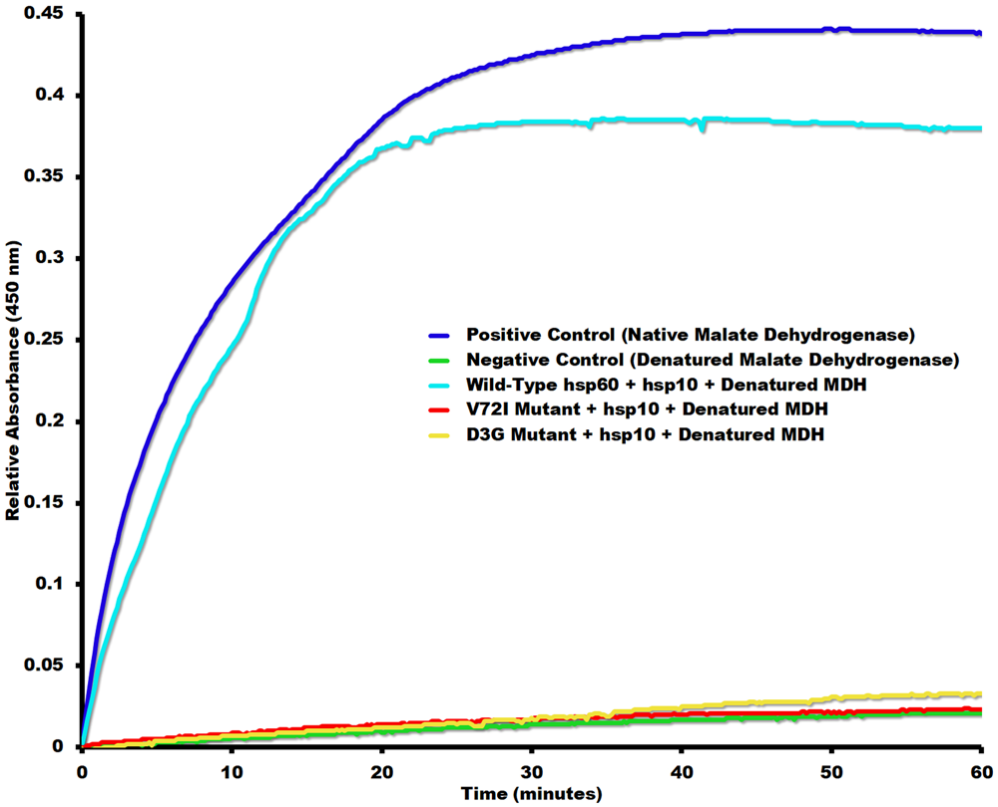
Malate Dehydrogenase Protein Folding Assay. This assay involves the refolding of acid-denatured malate dehydrogenase by ***wild***-***type*** hsp60/hsp10, *V72I* mutant/hsp10, **D3G** mutant/hsp10 . The assay conditions are described in “Methods” section. The positive control is native malate dehydrogenase (MDH) and its activity is determined by generating a product with absorbance at 450 nm that is proportional to the enzymatic activity present. The **negative control** consists of denatured malate dehydrogenase in the absence of chaperonin.

**Figure 7:**
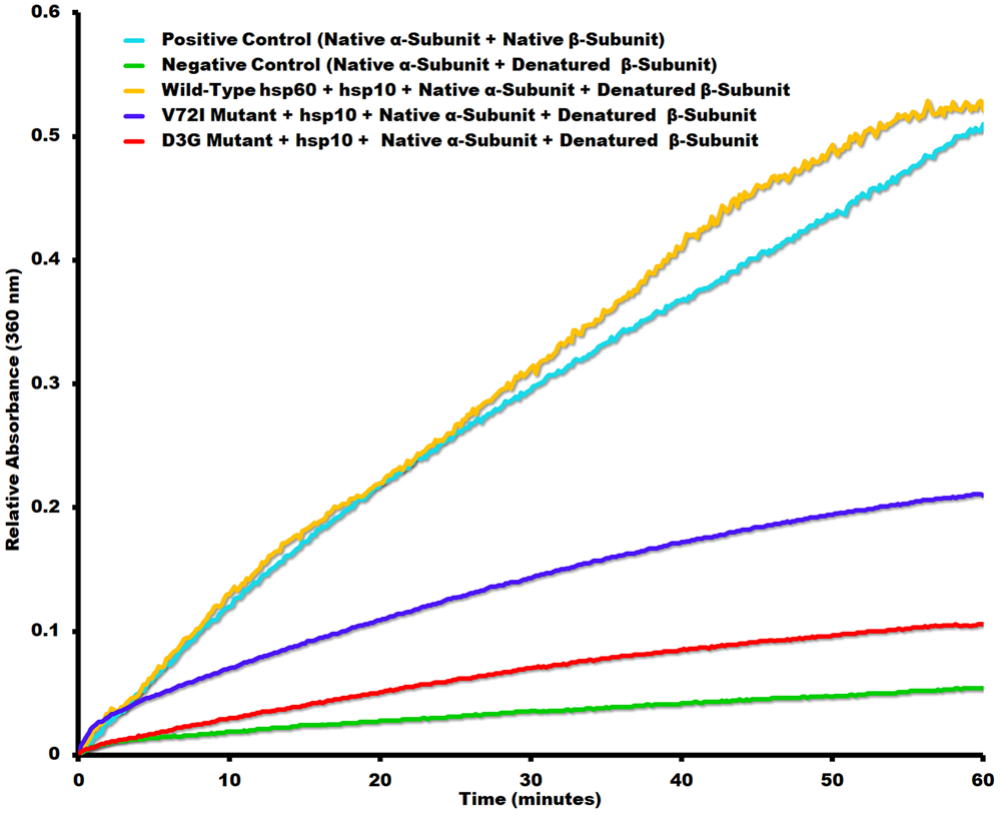
ATP synthase F_1_ β-Subunit Protein Folding Assay. The activity of native, thermally denatured, and refolded F_1_ ATP synthase β-subunit by chaperonin was detected using the EnzChek phosphate assay. The denatured β-Subunit in the absence of chaperonin served as a **negative control**, while the native β-Subunit in the absence of chaperonin served as a ***positive control***. In the presence of α-Subunit, refolding of denatured β-Subunit by **wild-type hsp60/hsp10**, V72I mutant/hsp10, *D3G mutant*/hsp10 was measured as described in the Methods section.

None of the proteins in this study were tagged to keep the proteins as close to wild type conditions as possible and before any biophysical experiments were to be performed, we verified that the proteins that were isolated were biologically active. Chaperonin protein-folding activity was measured using denatured α-lactalbumin as substrate previously used with groEL protein folding assays (Fig. 2)^27,37,38^. Mitochondrial specific substrates and disease related mutants were used to evaluate their protein-folding activity. Wild type hsp60/10 was able to effectively refold denatured malate dehydrogenase while the mutants D3G and V72I were completely inactive (Fig. 6), which is consistent with previous studies^17,19^. The β-subunit of the human mitochondrial ATP synthase has been shown to co-immunoprecipitate with hsp60 raising the possibility that the β-subunit is likely being folded by the chaperonin. Therefore, we tested if the β-subunit of the mitochondrial ATP synthase was a substrate of hsp60. The assay was repeated with the denatured ATP synthase β-subunit as substrate resulting in a significant decrease in refolding activity (Fig. 7). This is the first study demonstrating that the β-subunit of the mitochondrial ATP synthase is a substrate of hsp60/10 chaperonin system. In addition, the mutant hsp60/10 complexes are deficient in their ability to fold the β-subunit.

### Structure validation

**Figure 8:**
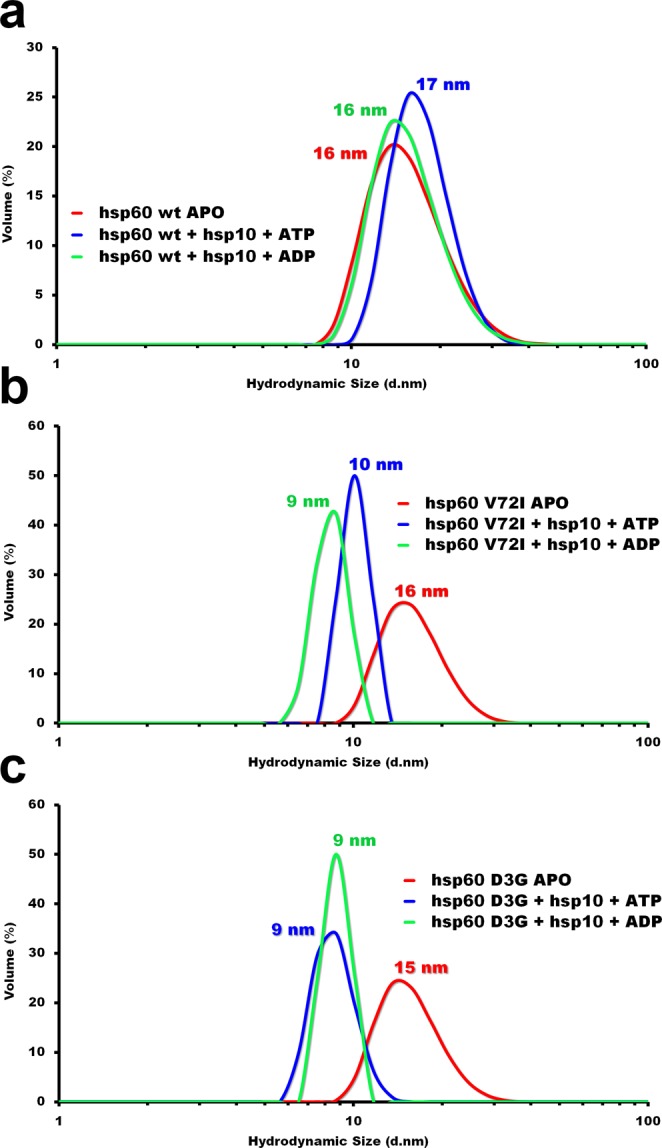
Dynamic light scattering reveals mutant hsp60 disassembly. (**a**) Dynamic light scattering (DLS) analysis of the hsp60 wildtype conformations in absence and presence of nucleotides. (**b**) Dynamic light scattering (DLS) analysis of the hsp60 V72I mutant conformations in absence and presence of nucleotides. (**c**) Dynamic light scattering (DLS) analysis of the hsp60 D3G conformations in absence and presence of nucleotides. Each curve reveals a different hydrodynamic diameter of the hsp60 chaperonin in the ATP bound (blue curve), ADP bound (cyan curve) and APO conformation (red curve).

To validate the negative-stain EM reconstructions, we performed dynamic light scattering (DLS) experiments on the wild type and mutant complexes to determine if the complexes were disassembling into monomers, Wild type hsp60/10 complexes remained in a tetradecameric conformation when nucleotide was added (Fig. 8a). The structure was slightly larger in the presence of ATP due to hsp10 binding at both ends of the complex. The D3G and V72I mutants on the other hand reveal an abrupt change in size upon the addition of nucleotide indicating a catastrophic event that led to the disassembly of the complex into monomers (Fig. 8b,c). The tetradecameric complex is about 16 nm in size while the monomer is expected to be about 9 nm based on the X-ray structure of one hsp60 subunit^35^.

## Discussion

Failure to acquire proper protein-folding to create normal tertiary or quaternary structure leads to impaired physiological functions and numerous disease conditions^39,40^. The working hypothesis during the studies presented here was that the diseases MitCHAP-60 and SPG13 were not directly related to the hsp60 mutations but instead were the consequence of an inactive hsp60/10 deficient in folding mitochondrial substrate proteins. Previous studies have suggested that these mutations impair the protein folding activity of hsp60 complexes, however, the detailed mechanism by which these mutations lead the neuromuscular diseases remains unknown. To study the effects of the D3G and V72I mutations and the underlying cause of MitCHAP-60 and SPG13, we first characterized the wild type chaperonin in terms of conformational changes that take place during the catalytic cycle.

Once we established that the purified proteins were biologically active, samples of the proteins were subjected to negative-stain electron microscopy to study the conformational states induced by various nucleotides and to determine if the mutations were affecting protein folding by altering the various conformational states. The APO conformation was determined previously in our lab and so we focused on the ATP and ADP conformations^27^. In previous studies in our lab, we were able to determine that the bacteriophage chaperonin φEL would not proceed along the protein-folding pathway in the absence of substrate and would instead stall at a conformation that is induced by either ATP, ADP or the absence of nucleotide (APO)^26^.

Processing of the negative stain data provided low resolution reconstructions that reveal dramatic conformational changes that take place after ATP binding, ATP hydrolysis, and after the removal of the ADP from the nucleotide binding pocket (Fig. 3). The addition of 2 mM ATP to hsp60/hsp10 resulted in a reconstruction of a tetradecameric hsp60 structure with the hsp10 co-chaperonin capping only one of the chaperonin protein-folding chambers resulting in a so-called “bullet” conformation (Fig. 3). The addition of 100 mM ATP prompted the formation of the “American football” conformation where the hsp10 co-chaperonin is bound to both openings to the protein-folding chambers. Just like what was seen with the φEL chaperonin, the addition of 2 mM ADP to mimic the hydrolyzed ATP conformation resulted in a single-ring conformation where the chaperonin separates at the equatorial domain to produce two C7 single-rings^41^. Even though several groups reported hsp60 ring separation, they never visualized single-rings in the presence of ADP. For example, Nielsen *et al*. reported that a single ring is enough for productive chaperonin-mediated folding *in-vivo*^25,42^. Instead, they used an hsp60 mutation to generate the single-ring conformation, while we used the wildtype hsp60 chaperonin and ADP to get ring separation. It was shown that the φEL separated into to single-rings to allow for the folding of viral proteins that could not be accommodated by the host chaperonin when the bacteriophage EL infected *Pseudomonas aeruginosa*. This was demonstrated to be true since the φEL chaperonin was able to refold the 116 kDa denatured β-galactosidase, restoring its activity to normal levels *in-vitro*^26^. It is unclear why the human hsp60/10 chaperonin utilizes the single-ring conformational intermediate since it is not able to refold β-galactosidase *in-vitro* (data not shown). Perhaps, it is required for the refolding of proteins not quite as large as β-galactosidase and may utilize a mechanism similar to the cytosolic TriC chaperonin where parts of the denatured substrate are folded sequentially^43^.

The resulting reconstructions have allowed us to postulate a likely catalytic pathway with various conformational intermediates (Fig. 4). The double-ring APO conformation is unable to bind hsp10 and therefore has accessible protein-folding chambers where a protein might be able to bind initially. Upon ATP binding, there is a conformational change that allows not only the internalization of the substrate but the simultaneous binding of the co-chaperonin hsp10. It is conceivable that after the binding of the first substrate and co-chaperonin, a conformational change may trigger the binding of substrate and co-chaperonin on the opposite hsp60 ring to form the double ring “American football” conformation seen by other groups^44^. ATP hydrolysis then triggers the separation of the two rings at the equatorial domain and the formation of the closed single-ring hsp60/10 conformation. Subsequent removal of the ADP from the nucleotide binding site triggers two single-rings to come back together to form the APO conformation and the re-initiation of the cycle. We would like to point out that we propose this model based on the conformations that we and others have observed under the electron microscope. One limitation of our model is that these structures were obtained in our study in the absence of substrate and therefore we cannot exclude the possibility of alternative conformational intermediates.

The negative-stain reconstructions allowed for a baseline model for how the human mitochondrial hsp60/10 chaperonin undergoes conformational changes during the protein-folding catalytic pathway (Fig. 4). Next, we proceeded with the characterization of the hsp60 D3G and V72I mutants by first purifying the mutant proteins and checking for protein folding activity using denatured α-lactalbumin as a substrate. Figure 2 illustrates how the V72I mutant had about a third of the activity of the wild type hsp-60 protein while the D3G mutant had only about one tenth the activity. These diminished levels of activity and their relative drop in activity appear to correlate with the severity of the diseases where D3G is more severe. Previous studies have reported diminished protein folding activity for the mutant complexes as well^17,19^. However, due to the labile nature of hsp60/10 protein complexes, they had to rely on reconstitution techniques to obtain tetradecameric rings. Our results confirm their findings, using denatured α-lactalbumin as a substrate, in a more biologically relevant system since we were able to purify fully assembled and functional hsp60/10 tetradecameric complexes.

We then proceeded to analyze the hsp60/10 complexes using electron microscopy since the effect of the D3G and V72I mutations had not been directly visualized in the presence and absence of nucleotides ATP and ADP. The purified mutant proteins were then applied to carbon coated electron microscopy grids for negative-stain data collection. Each sample was treated like the wild type with ATP, ADP and no nucleotide to produce the APO conformation. The results were very surprising in that only the untreated APO samples resulted in useable data. D3G and V72I mutant hsp60 samples treated with either ATP or ADP clearly fell apart into monomers (Fig. 5). The experiment was repeated an exhaustive number of times but yielded the same results consistently. Attempts at optimizing the conditions to favor a more stable structure all failed. We arrived at the conclusion that the mutant hsp60 tetradecameric complexes can be readily formed by self-assembly but then likely fail to undergo the requisite conformational changes required by either ATP or ADP binding and the complexes instead fall apart. This would also explain the loss of activity seen in the denatured α-lactalbumin refolding experiment (Fig. 2). Parnas *et al*. 2009 had previously reported that the D3G mutation leads to destabilization of the reconstituted hsp60 complexes at lower protein concentrations using crosslinking experiments. However, they reported that addition of 2 mM ATP in the crosslinking reactions increased the oligomeric forms, including tetradecamers. In contrast, our electron microscopy results demonstrate that the D3G and V72I mutant hsp60/10 protein complexes fell apart when treated with either ATP or ADP. These differences could be due to the purification or crosslinking experimental conditions. Our results were confirmed in dynamic light scattering experiments where the tetradecameric complex is detected by the instrument at a hydrodynamic size of about 16 nm. In the presence of nucleotide (ATP or ADP), the complex at 16 nm disappears and a peak reappears at about 9 nm. It must be noted that the location of the D3G and V72I mutations are near the ATP binding site and so it is likely that the mutations are interfering with the conformational changes that occur upon ATP binding and its subsequent hydrolysis.

The loss of hsp60/10 activity leads to neurodegeneration but this loss of activity cannot be a direct cause of the symptoms displayed in the patients of these disorders. A more likely scenario is that a mitochondrial protein that is a substrate for hsp60/10 is no longer folded correctly and this in turn leads to neurodegeneration^45^. In fact, hsp60/10 has been shown to be involved in assembly of the mitochondrial ATP synthase complex^46–48^. It would not be surprising if hsp60/10 activity is decreased resulting in an altered ATP synthase activity, decreased mitochondrial function, affecting the muscle and nerve tissues the most because that is where ATP is in highest demand.

The mitochondrial ATP synthase is a multi-subunit enzyme composed of two domains, F_O_ and F_1_, which together with other proteins function as a rotational motor to allow for ATP production during respiration^49,50^. The F_1_ domain of the ATP synthase is responsible for the synthesis of ATP, where the α- and β-subunits form a catalytically active hexameric α_3_β_3_ complex^51^. The α_3_β_3_ complex, in isolation, hydrolyzes ATP instead of synthesizing it. The D3G mutation near the hsp60 amino terminus is thought to result in the loss of function where the D3G-hsp60 disassembles by entropic destabilization^17,52^. Our work now builds on this idea by adding that the entropic destabilization occurs through the inability to undergo normal conformational changes and instead result in disassembly of the chaperonin. This suggests that non-functional hsp60/10 complexes would be unable to assemble the ATP synthase, resulting in abnormal production of ATP inside the mitochondria. In addition, hsp60/10 and the ATP synthase β-subunit, together, have been shown to be differentially expressed in breast cancer, null cell pituitary adenoma, Barrett’s esophagus (premalignant condition to esophageal adenocarcinoma), and head and neck squamous cell carcinomas^53–56^. This concomitant regulation suggests that hsp60/10 and the ATP synthase β-subunit work closely together in maintaining the cellular oxidative phosphorylation. Defects that result in functional impairment of the mitochondrial ATP synthase have been shown to cause a variety of neuromuscular disorders (reviewed in^57^). Likewise, defects in mitochondrial function have also been linked to various neurodegenerative diseases (reviewed in^58^ and^59^). This led us to posit that mutations in hsp60 lead to SPG13 and MitCHAP-60 via an inability to fold the ATP synthase β-subunit and other mitochondrial proteins such as malate dehydrogenase that are part of the reactions of aerobic respiration that culminate in ATP synthesis.

The purified and reconstituted α and β subunits of the ATPase synthase self-assemble into a α_3_β_3_ complex that possess ATPase activity *in-vitro*. To investigate the chaperonin activity of the hsp60/10 complexes, we denatured the β-subunit, and measured the ability of the hsp60/10 complexes to refold the β-subunit. Renatured β-subunit would form a complex with the native α-subunit leading to functional complexes with ATPase activity that can be measured via the EnzChek phosphatase assay kit. The assay measures inorganic phosphate released from two sources, the hsp60/10 complexes catalyzing the refolding of ATP synthase β-subunit and the functional α-β ATPase synthase complexes themselves (after renaturation of the β-subunit). The α-β ATPase synthase complexes however hydrolyze an excess of ATP that outcompetes that of the chaperonin. The assays in described in Figs 6 and 7 confirm that the wild-type hsp60/10 can refold mitochondrial proteins involved in aerobic respiration including malate dehydrogenase and the β-subunit of the ATP synthase. SPG13 (V72I) doesn’t result in death of human patients because there is some residual activity as seen in Fig. 7 where activity is approximately 40% of the wild-type chaperonin activity when refolding the ATP synthase β-subunit. MitChap-60 (D3G) on the other hand has about 20% of the wild-type chaperonin activity and results in symptoms that are more severe and eventual patient death. This trend is consistent with data obtained with denatured α-lactalbumin as substrate as seen in Fig. 2 where SPG13 had 35% of wild-type activity and MitChap-60 had 12% of wild-type activity. Previous studies have reported a reduction in the catalytic activity of D3G and V71I mutant complexes using substrate MDH. We confirm their biochemical findings using a more biologically relevant hsp60/10 system using a number of folding substrates. In addition, this is the first study demonstrating that the β-subunit of the mitochondrial ATP synthase is a substrate of hsp60/10 chaperonin system and that the mutant hsp60/10 complexes are deficient in their ability to fold the β-subunit.

## Conclusions

Our data suggests that hsp60/10 plays a crucial role in folding important mitochondrial proteins involved in aerobic respiration including the β-subunit of the ATP synthase and malate dehydrogenase^45^. D3G and V72I point mutations in hsp60 impair the chaperonin’s ability to fold these substrates. This in turn may lead to abnormal ATP synthesis where the diminished ATP levels would severely impact muscle and neuronal cells which have high energy demands. This in turn may lead to the MitCHAP60 and PSG13 neurodegenerative disorders.

## Data Availability

All data and reagents could be made available from the corresponding authors upon request.
